# Analysis of nucleic acids extracted from rapid diagnostic tests reveals a significant proportion of false positive test results associated with recent malaria treatment

**DOI:** 10.1186/s12936-022-04043-7

**Published:** 2022-01-24

**Authors:** Salome Hosch, Charlene Aya Yoboue, Olivier Tresor Donfack, Etienne A. Guirou, Jean-Pierre Dangy, Maxmillian Mpina, Elizabeth Nyakurungu, Koranan Blöchliger, Carlos A. Guerra, Wonder P. Phiri, Mitoha Ondo’o Ayekaba, Guillermo A. García, Marcel Tanner, Claudia Daubenberger, Tobias Schindler

**Affiliations:** 1 Swiss Tropical and Public Health Institute, Basel, Switzerland; 2 University of Basel, Basel, Switzerland; 3 Medical Care Development International, Malabo, Equatorial Guinea; 4 Ifakara Health Institute, Bagamoyo, United Republic of Tanzania; 5 Laboratorio de Investigaciones de Baney, Baney, Equatorial Guinea; 6 Ministry of Health and Social Welfare, Malabo, Equatorial Guinea; 7 Centre Suisse de Recherches Scientifiques en Côte d’Ivoire, Abidjan, Côte d’Ivoire

**Keywords:** Molecular malaria surveillance, False-positive malaria rapid diagnostic test, PfHRP2 persistence, *pfhrp2* gene deletion

## Abstract

**Background:**

Surveillance programmes often use malaria rapid diagnostic tests (RDTs) to determine the proportion of the population carrying parasites in their peripheral blood to assess the malaria transmission intensity. Despite an increasing number of reports on false-negative and false-positive RDT results, there is a lack of systematic quality control activities for RDTs deployed in malaria surveillance programmes.

**Methods:**

The diagnostic performance of field-deployed RDTs used for malaria surveys was assessed by retrospective molecular analysis of the blood retained on the tests.

**Results:**

Of the 2865 RDTs that were collected in 2018 on Bioko Island and analysed in this study, 4.7% had a false-negative result. These false-negative RDTs were associated with low parasite density infections. In 16.6% of analysed samples, masked *pfhrp2* and *pfhrp3* gene deletions were identified, in which at least one *Plasmodium falciparum* strain carried a gene deletion. Among all positive RDTs analysed, 28.4% were tested negative by qPCR and therefore considered to be false-positive. Analysing the questionnaire data collected from the participants, this high proportion of false-positive RDTs could be explained by *P. falciparum* histidine rich protein 2 (PfHRP2) antigen persistence after recent malaria treatment.

**Conclusion:**

Malaria surveillance depending solely on RDTs needs well-integrated quality control procedures to assess the extent and impact of reduced sensitivity and specificity of RDTs on malaria control programmes.

**Supplementary Information:**

The online version contains supplementary material available at 10.1186/s12936-022-04043-7.

## Background

According to the World Health Organization (WHO), more than 409,000 malaria-related deaths were reported in 2019, most of them in children below the age of 5 years [1]. The majority of malaria infections (94%) and malaria-related deaths (95%) occurred in sub-Saharan Africa (SSA) [2], where *Plasmodium falciparum* is the dominant malaria parasite [1]. The test-treat-track strategy advised by WHO is one of the backbones of current malaria control and elimination programmes [3]. This strategy entails every suspected malaria case be tested, every confirmed case be treated, and the disease be tracked through surveillance systems [4]. Testing relies heavily on rapid diagnostic tests (RDTs), exemplified by the more than 348 million RDTs distributed globally in 2019 [1]. In SSA, RDTs have almost completely replaced light microscopy for malaria diagnosis, accounting for an estimated 75% of all malaria tests conducted in 2017 [5]. RDTs are point-of-care tests that detect circulating antigens, such as the *P. falciparum*-specific histidine rich protein 2 (PfHRP2) or histidine rich protein 3 (PfHRP3), as well as the pan-*Plasmodium* spp. enzymes, lactate dehydrogenase (pLDH) or aldolase [6]. More than 90% of RDTs currently in use target the PfHRP2 antigen because of its higher sensitivity compared to non-PfHRP2 antigens [7]. PfHRP2-based RDTs used for the diagnosis of febrile patients that suffer from malaria infection are highly sensitive and specific [8]. RDTs are often used by national malaria surveillance programmes. However, when individuals are asymptomatic with low parasite densities, RDTs often fail to detect the parasites due to low antigen concentrations [9, 10].

A recent study showed that false-negative RDTs (FN-RDT) are more common in lower malaria transmission settings, younger subjects and in urban areas in SSA [11]. Reduced diagnostic performance of RDTs has also been attributed to genetic diversity of the *pfhrp2* gene [12], differences in expression levels of PfHRP2 antigen in parasite field strains [13], or deletion of *pfhrp2* and *pfhrp3* genes in isolates [14]. *Pfhrp2* gene deletions appear to be common and therefore are relevant as they might be a threat to malaria control programmes based on monitoring of malaria prevalence through RDTs [15, 16].

Less attention has been given to the specificity of malaria RDTs used in malaria surveys that potentially result in false-positive results. False-positive RDTs (FP-RDT) have been associated with high levels of circulating rheumatoid factor [17–19] or acute typhoid fever [20]. There is evidence of FP-RDTs in patients infected with *Schistosoma mekongi* [21] or human African trypanosomiasis [22]. FP-RDTs are also caused by persisting antigen circulation in peripheral blood after successful *P. falciparum* drug treatment. A meta-analysis revealed that around half of the PfHRP2-detecting RDTs remain positive 15 days (95% CI 5–32) post *P. falciparum* treatment, which is 13 days longer than RDTs based on the pLDH antigen [23]. The latter study also reported a higher persistent RDT positivity among individuals treated with artemisinin combination therapy (ACT) than those treated with other anti-malarial drugs. Since RDTs are instrumental to malaria surveillance programmes, their diagnostic performance should be systematically monitored over time using sensitive and highly specific methods detecting *Plasmodium* spp. molecular markers. Described here is an approach for quality control of field-deployed RDTs by retrospective molecular analysis of the parasite DNA retained on them using RT-qPCR.

## Methods

### The 2018 malaria indicator survey conducted on Bioko Island as a biobank of RDTs for molecular malaria surveillance

A malaria indicator survey (MIS) has been conducted annually since 2004 on the island of Bioko, Equatorial Guinea, to evaluate the impact of malaria control interventions [24]. The survey uses a standard questionnaire developed by the Roll Back Malaria initiative to gather information on selected households and their occupants. The 2018 Bioko Island MIS covered 4774 households with 20,012 permanent residents, among whom 13,505 persons consented to storage and molecular analysis of their RDT. Briefly, consenting individuals living in surveyed households are tested for malaria and malaria-related anaemia. Malaria testing was done with the CareStart™ Malaria HRP2/pLDH (Pf/PAN) combo test (ACCESS BIO, NJ, USA). PfHRP2-positive RDTs were recorded as *P. falciparum*, pLDH-positive RDTs as *Plasmodium* spp. and RDT-positive for both antigens as mixed. The haemoglobin level in peripheral blood was measured during the MIS using a battery-operated portable HemoCue system (HemoCue AB, Ängelholm, Sweden). The anaemia status (mild, moderate, severe) was categorized based on definitions published by WHO [25] stratified by age, gender and pregnancy status. Households were assigned scores based on the type of assets and amenities they own to derive a surrogate of their socio-economic status (SES), using principal component analysis (PCA). After ranking all households based on their score, they were divided into five equal categories (quintiles), each with approximately 20% of the households. The first quintile corresponded to the lowest wealth index and the fifth to the highest wealth index. The household wealth index categories were also assigned to permanent household members.

### Detection and quantification of *Plasmodium* spp. nucleic acids extracted from RDTs

A previously published dataset generated with the Extraction of Nucleic Acids from RDTs (ENAR) protocol developed by the authors was extended for this study [26]. Briefly, RDTs were barcoded, stored at room temperature and shipped to Basel, Switzerland, for nucleic acid (NA) extraction and detection. This approach simplifies small volume blood collection, transport and storage logistics, and allows linking outcomes of molecular-based detection of parasite-derived NA with the demographic and socio-economic information collected from each corresponding MIS participant at high throughput.

All 2865 samples were initially screened with the PlasQ RT-qPCR assay [27]. In this RT-qPCR assay, the high copy number *P. falciparum*-specific varATS region [28] and the pan-*Plasmodium* 18S rDNA gene were targeted [29, 30]. Samples with cycle of quantification (Cq) value < 45 in two replicates of either of the two targets, varATS or 18S rDNA, were considered positive for active blood-stage malaria infection. *Plasmodium falciparum* parasites were quantified based on their Cq value for varATS [26]. In addition, only samples with Cq value < 35 for amplification of the internal control gene, the human *rnasep* gene were included, to demonstrate that the NA extracted from the RDTs is sufficient for reliable molecular analysis of malaria parasites. Non-*falciparum* malaria species identification of samples positive for the pan-*Plasmodium* target 18S rDNA was performed with a multiplex RT-qPCR assay based on species-specific 18S rDNA sequences as described previously [26].

### Quality control and categorization of RDT outcomes

A RDT was considered positive if a healthcare worker recorded a positive signal for the PfHRP2, pLDH or both targets during the MIS. Among these positive RDTs, a true-positive RDT (TP-RDT) result was defined as a RDT with detectable *Plasmodium* spp. NA (two replicates with varATS and/or 18S rDNA Cq < 45 and human *rnasep* Cq < 35). A FP-RDT result was defined as positively read and recorded RDT in the field but with a negative outcome for *Plasmodium* spp. NA based on PlasQ RT-qPCR in the presence of human *rnasep* Cq < 35. Negative RDTs were classified as being read as negative by a healthcare worker during the MIS and recorded in the database. A true-negative RDT (TN-RDT) result was defined as a RDT whose negative result collected in the field was confirmed by the PlasQ RT-qPCR. A FN-RDT result was defined as negatively read by a healthcare worker in the field with a positive PlasQ RT-qPCR result based on two replicate amplifications with varATS and/or 18S rDNA Cq < 45 and the human *rnasep* Cq < 35.

### qHRP2/3-del assay for detection of *pfhrp2* and *pfhrp3* deletions

The previously published qHRP2/3-del assay that simultaneously amplifies the *pfhrp2* and *pfhrp3* genes together with the internal control gene *pfrnr2e2* was adapted to accommodate for the lower input of NA [31]. Briefly, the probe for the internal control gene *pfrnr2e2* was labelled with fluorescein (FAM) instead of Cy5 to improve its detectability. Additionally, the final concentration of all primers was increased from 0.3 µM to 0.45 µM. Concentrations of 0.15 µM were used for the *pfrnr2e2* probe, and 0.225 µM for the *pfhrp2* and *pfhrp3* probes each. All samples were run in triplicates and the number of amplification cycles was increased from 45 to 50. Every 96-well qPCR plate contained control DNA extracted from a known *pfhrp2*-deleted *P. falciparum* strain (Dd2), a *pfhrp3*-deleted *P. falciparum* strain (HB3), and a *P. falciparum* strain without *pfhrp2* and *pfhrp3* gene deletions (NF54) as well as a non-template control (NTC). Successful amplification was defined as a mean Cq < 40 for *pfrnr2e2* calculated from at least two replicates for each sample. The qHRP2/3-del assay only was run with NA extracted from RDTs that had displayed a Cq < 35 for the *varATS* target in the PlasQ RT-qPCR.

*Pfrnr2e2*, *pfhrp2* and *pfhrp3* are all single-copy genes and they show comparable performances in the multiplex qPCR assay [31]. One approach to detect *P. falciparum* strains with *pfhrp2* and/or *pfhrp3* gene deletions in mixed *P. falciparum* strain infections (herein defined as masked gene deletions), is to calculate the difference in Cq values obtained between *pfhrp2* or *pfhrp3* and *pfrnr2e2* amplifications (ΔCq values). This is done by subtracting the Cq value obtained during the amplification of *pfrnr2e2* from the Cq value of *pfhrp2* or *pfhrp3*, respectively. Combining all runs that were conducted, the mean ΔCq for *pfhrp2* in controls (NF54 and HB3) was 0.00 (SD ± 0.52) and for *pfhrp3* the mean ΔCq in controls (NF54 and Dd2) was 1.19 (SD ± 0.83). For *pfhrp2* the ΔCq cut-off value of 2.0 determined by Schindler et al. [31] was used to identify masked gene deletions. For *pfhrp3* a ΔCq cut-off value of 4.0 was chosen to identify masked gene deletions due to the shift in mean ΔCq in the controls.

### Genotyping of *Plasmodium falciparum pfmsp1 *and *pfmsp2 genes*

Genotyping with *pfmsp1* and *pfmsp2* was performed following published procedures using nested PCR [32]. The first two PCR reactions amplify conserved sequences within the polymorphic regions of *pfmsp1* and *pfmsp2*, respectively. The second, nested PCR targets allele-specific sequences in five separate reactions. Samples were run in 20-µL total volume with 1 × Hot Firepol Master Mix (Solys BioDyne, Estonia), 0.25 µM of forward and reverse primers and 2-µL template DNA. The cycling conditions for the first PCR were 95 °C for 12 min, 25 cycles of 95 °C for 30 s, 58 °C for 1 min and 72 °C for 2 min and 72 °C for 10 min. For the second PCR, the cycling conditions for the three allele-specific *pfmsp1* primer pairs were 95 °C for 12 min, 35 cycles of 95 °C for 30 s, 56 °C for 40 s and 72 °C for 40 s and 72 °C for 10 min. For the two *pfmsp2* allele-specific reactions the conditions were: 95 °C for 12 min, 35 cycles of 95 °C for 30 s, 58 °C for 40 s and 72 °C for 40 s and 72 °C for 10 min. Presence and size of PCR products was determined and documented visually on a 1% agarose gel with a 100 bp DNA ladder.

### Genotyping of *Plasmodium malariae* circumsporozoite protein (pmcsp)

The *pmcsp* gene was amplified by semi-nested PCR for all samples with a positive signal for *Plasmodium malariae* in the non-*falciparum* malaria species identification assay [26]. The first PCR was run with 3 μL of DNA template in a reaction volume of 20 μL. The reaction mix contained 1 × Hot Firepol Master Mix and 0.25 μM of each of the primers csp_OF [33] and csp-R [34]. The conditions for the first PCR were: 95 °C for 12 min; 35 cycles of 95 °C for 15 s, 53 °C for 30 s and 65 °C for 90 s and final elongation at 65 °C for 10 min. The second, semi-nested PCR used 1.5 μL of the product from the first reaction in a total volume of 15 μL. The reaction mix contained 1 × Hot Firepol Master Mix and 0.33 μM of the primers csp_IF [33] and csp-R. The conditions for the second PCR were: 95 °C for 12 min; 35 cycles of 95 °C for 15 s, 52 °C for 30 s and 62 °C for 90 s and final elongation at 62 °C for 10 min. The PCR product was sent to Microsynth (Microsynth AG, Switzerland) for bidirectional sanger sequencing. The 15 sequences of *P. malariae* circumsporozoite protein from Bioko Island have been deposited into GenBank under the Accession Numbers MW963324–MW963338.

### Data analysis and statistics

The generated (RT)-qPCR data was initially analysed with the CFX Maestro Software (Bio-Rad Laboratories, CA, USA). Thresholds for each fluorescence channel were set manually and Cq values were then uploaded to the ELIMU-MDx platform for data storage and analysis [23]. Sequence analysis was performed using Geneious Prime 2019.1.1 (https://www.geneious.com). Statistical analysis and data visualization was performed using the R statistical language (version 4.0.3) based on packages data.table, dplyr, epiDisplay, epitools, ggplot2, ggpubr, ggridges, gridExtra, lme4, readxl, reshape2, scales, stringr, tidyr, tidyverse. Wilcoxon rank sum test was used for numeric values. Fisher’s exact test (two-sided) was used for contingency tables. A generalized linear mixed-effects model with fixed and random effects was used for calculation of odds ratios and their confidence intervals.

## Results

### Integration of molecular diagnostic methods into the national malaria control programme to assess the performance of malaria RDTs

In total, 2865 RDTs (21.2%) collected during the 2018 MIS were included in this study. The median age of volunteers included in this sample collection was 22 years (interquartile range 9 to 38 years), female participants were over-represented (58.2%), and 97.8% of the participants were asymptomatic, non-febrile individuals. Of the 507 (17.7%) participants that reported to have been sick in the 2 weeks preceding the survey, 81.5% (413/507) had had fever. Other common symptoms were headache in 34.1% (173/507), followed by articular and bone pain in 21.3% (108/507), pallor and weakness in 13.0% (66/507), vomiting in 11.9% (60/507), and shaking chills in 7.5% (38/507). Fever was accompanied by other symptoms in 87.2% (360/413) of those who reported to have had fever. More than two-thirds of the RDTs were collected in the urban areas of the capital city Malabo on Bioko Island.

**Fig. 1:**
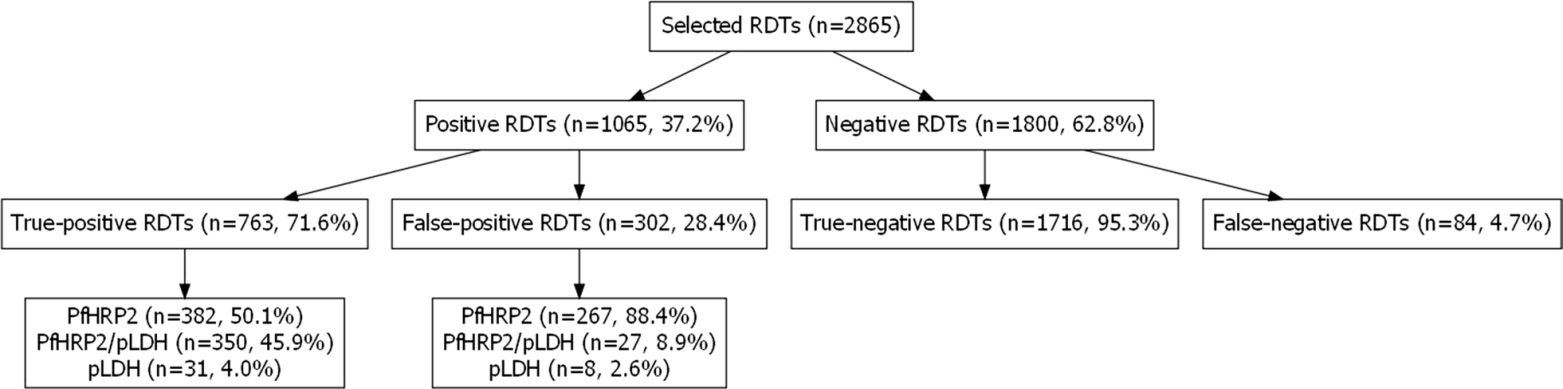
Comparison of rapid diagnostic test outcomes with PlasQ RT-qPCR results obtained after nucleic acid extraction and amplification. Nucleic acids were extracted from 2865 RDTs collected during the 2018 MIS and subsequently amplified with the PlasQ RT-qPCR to detect *Plasmodium* specific nucleic acids

Following NA extraction, a PlasQ RT-qPCR result was generated for 1800 malaria negative and 1065 positive RDTs, as recorded in the MIS database. By comparison between PlasQ RT-qPCR results with the RDT results collected in the field, RDTs were grouped into four categories, namely true-positive (TP), true-negative (TN), false-positive (FP), and false-negative (FN), respectively (Fig. 1). The PlasQ RT-qPCR was used as a gold standard to evaluate the performance of the RDT, and this resulted in an overall sensitivity of 90.0% and specificity of 85.0% of field-deployed RDTs.

When stratified by type of antigen, RDTs classified as FP-RDTs were predominantly those that detected the PfHRP2 antigen (88.4%); whereas 8.9 and 2.6% of the FP-RDTs were those that detected both PfHRP2 plus pLDH antigens or the pLDH antigen only, respectively. Around half of RDTs classified as TP-RDTs were those that detected the PfHRP2 antigen only (50.1%), followed by those that detected PfHRP2 plus pLDH antigens (45.9%) and lastly, those that detected the pLDH antigen only (4.0%).

### Low parasite density infections are likely to cause FP-RDT results in the field

**Fig. 2:**
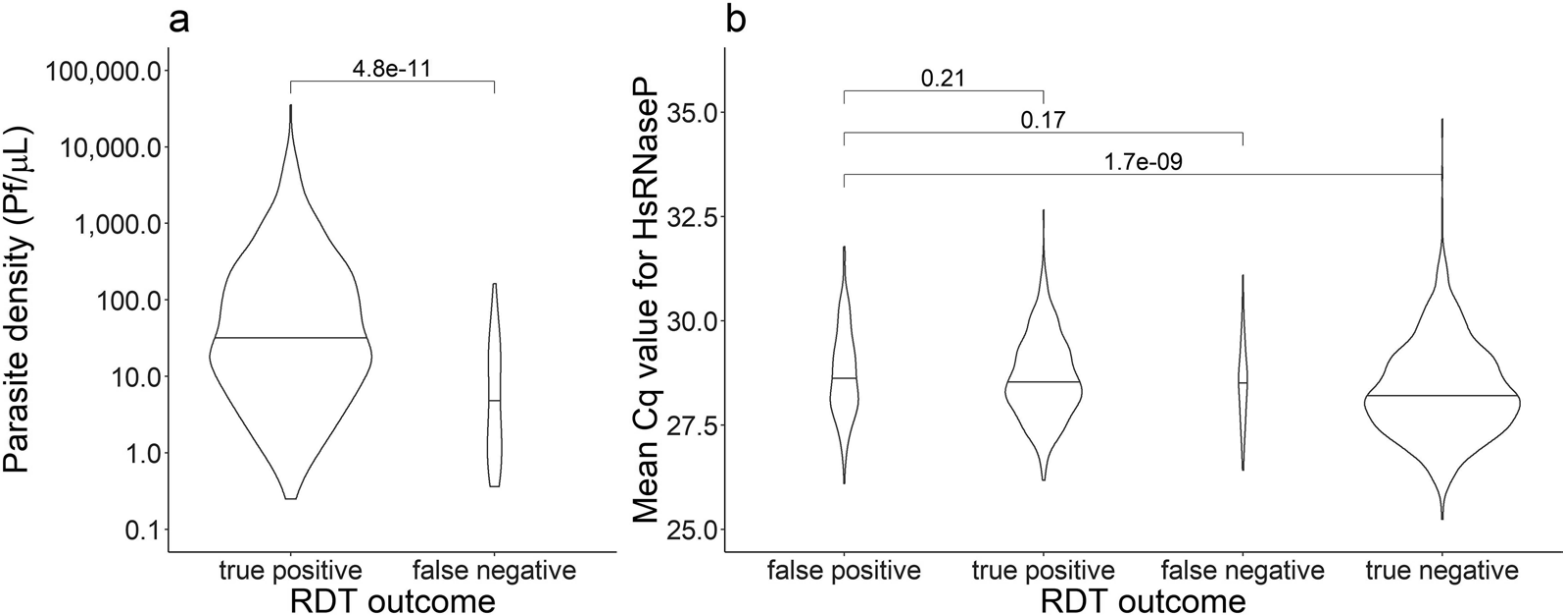
Rapid diagnostic test outcome in relation to *Plasmodium falciparum* parasite densities and qPCR amplification of human *rnasep* endogenous control. **a** *Plasmodium falciparum* parasite densities compared between true positive and false negative RDT outcomes. Parasite densities for *P. falciparum* were estimated based on the varATS target of the PlasQ RT-qPCR assay. Wilcoxon rank sum test was used to compare the two groups. **b** Comparison of the Cq values obtained with the amplification of the human *rnasep* gene used as internal control of the PlasQ RT-qPCR assay, across all samples stratified by RDT outcome. The group of RDTs with a false-positive result was compared to the other RDT outcomes by Wilcoxon rank sum test

The ENAR approach used in this study detects 10–100 times lower asexual blood stage parasite densities than the PfHRP2-based RDT [26]. The data confirms that a clear association exists between FN-RDT, TP-RDT and *P. falciparum* parasite densities assessed by the PlasQ RT-qPCR outcome. TP-RDT had higher geometric mean parasite densities (35.0 *P. falciparum*/µL, IQR: 7.2–166.0) compared to FN-RDTs (4.6 *P. falciparum*/µL, IQR: 1.1–20.0) (Fig. 2a, Wilcoxon rank sum test, p < 0.001). Although *P. falciparum* was the most common (93.8%) *Plasmodium* spp. species among RT-qPCR positive RDTs, *P. malariae* (4.0%) and *Plasmodium ovale* spp. (1.1%) were also identified. Co-infections between *Plasmodium* spp. species were included in these prevalence calculations. In 3.2% (27/847) of *Plasmodium* spp.-positive samples, no species could be assigned, possibly due to low parasite density and the generally lower sensitivity of the species-specific qPCR assays. No *Plasmodium vivax* and *Plasmodium knowlesi* parasite NAs were detected. The central repeat region of the *P. malariae* circumsporozoite protein (*pmcsp*) was amplified by PCR and Sanger sequenced to reconfirm the presence of *P. malariae* derived NA (Additional file 1: Fig. S1b). Nucleotide sequences were unique among all the 15 *P. malariae* PCR fragments sequenced and also the number of NAAG and NDAG repeats varied between these isolates indicating high diversity of the local *P. malariae* population. *Plasmodium malariae* was found among 6.6% of FN-RDTs compared to 3.8% among TP-RDTs. Similarly, *P. ovale* spp. was more prevalent in FN-RDTs (2.6%) than in TP-RDTs (0.9%).

To exclude the possibility that FP-RDTs are the consequence of failed amplification related to the degradation of NA retained on the RDTs, an additional analysis was carried out. During the PlasQ RT-qPCR, the human *rnasep* gene was used as an internal control to monitor the amount of NA extracted from each RDT. On average, the human *rnasep* was amplified with a Cq value of 28.5 (SD ± 1.0). There was no significant difference in the Cq values of the human *rnasep* gene amplification among RDTs, which were categorized as FP (28.6, SD ± 1.0), TP (28.5, SD ± 1.0), or FN (28.6, SD ± 1.0). TN-RDTs had a significantly lower median Cq value (28.2, SD ± 1.1) (Fig. 2b). These results indicate that the lack of detectable *P. falciparum* NA in the blood retained on FP-RDTs is not related to poor NA extraction performance or a failure in detecting NAs.

### FN-RDT results are not associated with parasites carrying *pfhrp2* and *pfhrp3* gene deletions

**Fig. 3:**
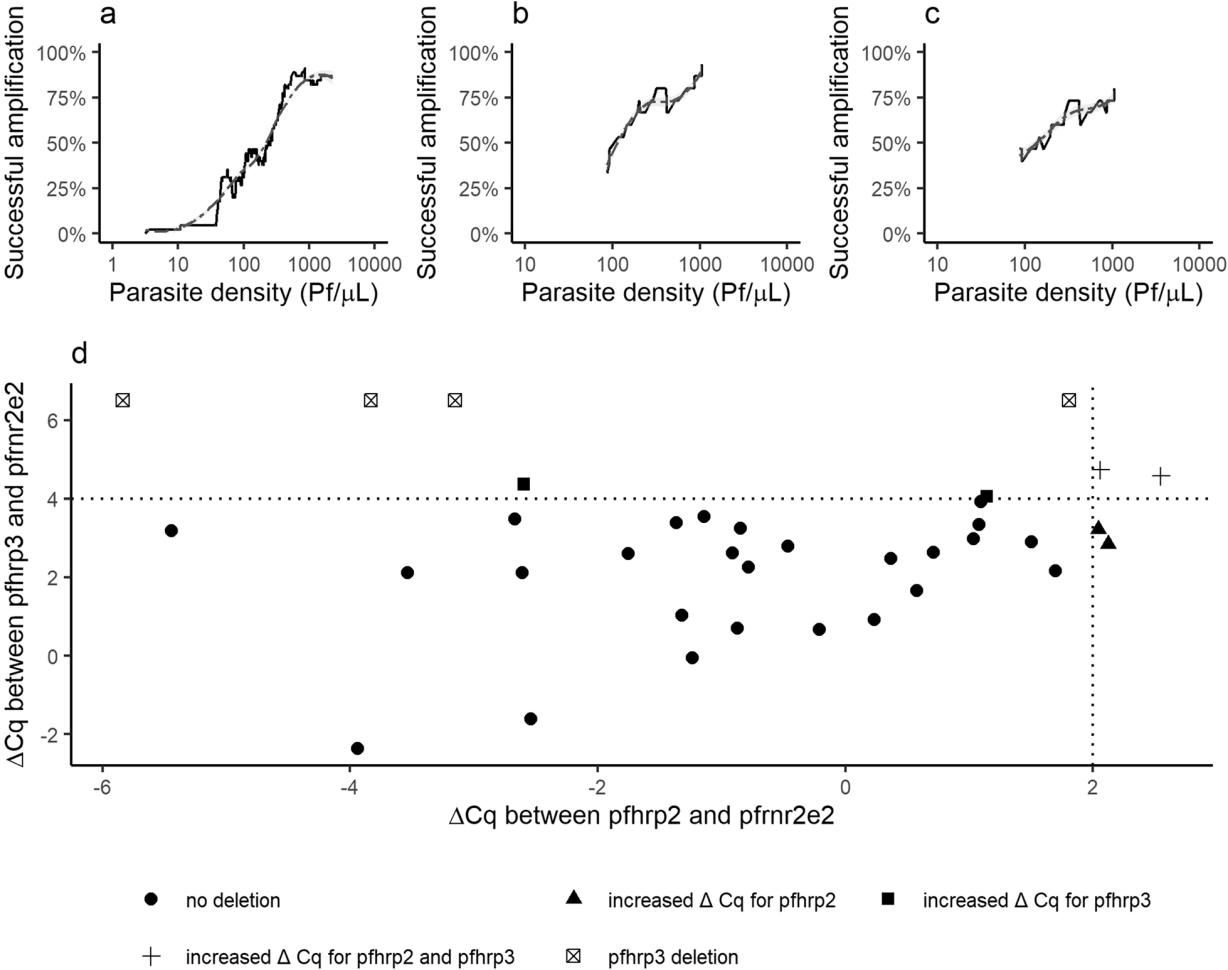
Amplification rate for three single copy genes and ΔCq values of the qHRP2/3-del assay. Amplification rate (rolling mean) for the genotyping reference genes **a** *pfrnr2e2*, **b** *pfmsp1* and **c** *pfmsp2* as a function of the parasite density of the sample. Parasite densities for *P. falciparum* were estimated based on the *varATS* target of the PlasQ assay. **d** The distribution of ΔCq values between *pfhrp2* (x-axis) or *pfhrp3* (y-axis) and *pfrnr2e2*. ΔCq thresholds (dashed lines) were set at 2.0 for *pfhrp2* and 4.0 for *pfhrp3*. To show the ΔCq for *pfhrp2* for samples with a *pfhrp3* deletion, the ΔCq for *pfhrp3* was set arbitrarily at 6.5

*Plasmodium falciparum* strains were genotyped to identify strains with *pfhrp2* and/or *pfhrp3* gene deletions. The number of samples available was limited based on the combination of low parasite density infections and the limited amount of blood retained on RDTs as a source of NA. The single copy gene *pfrnr2e2*, serving as the internal control of the qHRP2/3-del assay, was amplified with Cq < 40 in 184/406 (45.3%) samples. To avoid false reporting of *pfhrp2* and/or *pfhrp3* gene deletions, the analysis was restricted to samples that had an additional successful amplification in either *pfmsp1* (32/47, 68.1%) or *pfmsp2* (31/47, 66.0%). No amplification in *pfmsp1* or *pfmsp2* was observed in 23.4% (11/47) of samples. Based on the available data from the 27 samples with successful *pfmsp1* and *pfmsp2* genotyping (Additional file 1: Fig. S1a), polyclonal infections consisting of two or more distinct *P. falciparum* clones were found in 63.0% (17/27) of samples. Association between parasite density and amplification of each of the three distinct reference genes (*pfrnr2e2, pfmsp1* or *pfmsp2*) is shown in Fig. 3a–c. At least two out of three reference genes were amplified in 36 samples, which were then included in the analysis of the *pfhrp2* and *pfhrp3* deletion status. No evidence for parasites carrying a *pfhrp2* gene deletion was found in these 36 samples, but 4 out of 36 samples (11.1%) were likely to carry *pfhrp3* gene deletions. All 4 samples with *pfhrp3* deletion were recorded as positive for PfHRP2 by RDT.

The qHRP2/3-del assay was used to identify *pfhrp2* and/or *pfhrp3* gene deletions in polyclonal *P. falciparum* infections by calculating the ΔCq values as the difference of Cq values between *pfhrp2 and pfhrp3* gene amplification and the *pfrnr2e2* internal control. Figure 3d shows the distribution of samples with their respective ΔCq values for *pfhrp2* and *pfhrp3.* Of the 36 samples included, 2 samples (5.6%) had increased ΔCq values for both genes, 2 samples (5.6%) only for the *pfhrp2* gene and 2 samples (5.6%) only for the *pfhrp3* gene, respectively. Importantly, all 36 samples, which were screened for *pfhrp2* and *pfhrp3* gene deletions, were positive for PfHRP2 by RDT. Three out of 6 samples with increased ΔCq values for *pfhrp2* and/or *pfhrp3* were successfully genotyped with *pfmsp1* and *pfmsp2*. Two genotypes were found in one sample with increased ΔCq value for *pfhrp2* and *pfhrp3* each and a single genotype in one sample with increased ΔCq value for *pfhrp3*.

### FP-RDT results are associated with recent use of anti-malarial drugs

**Fig. 4:**
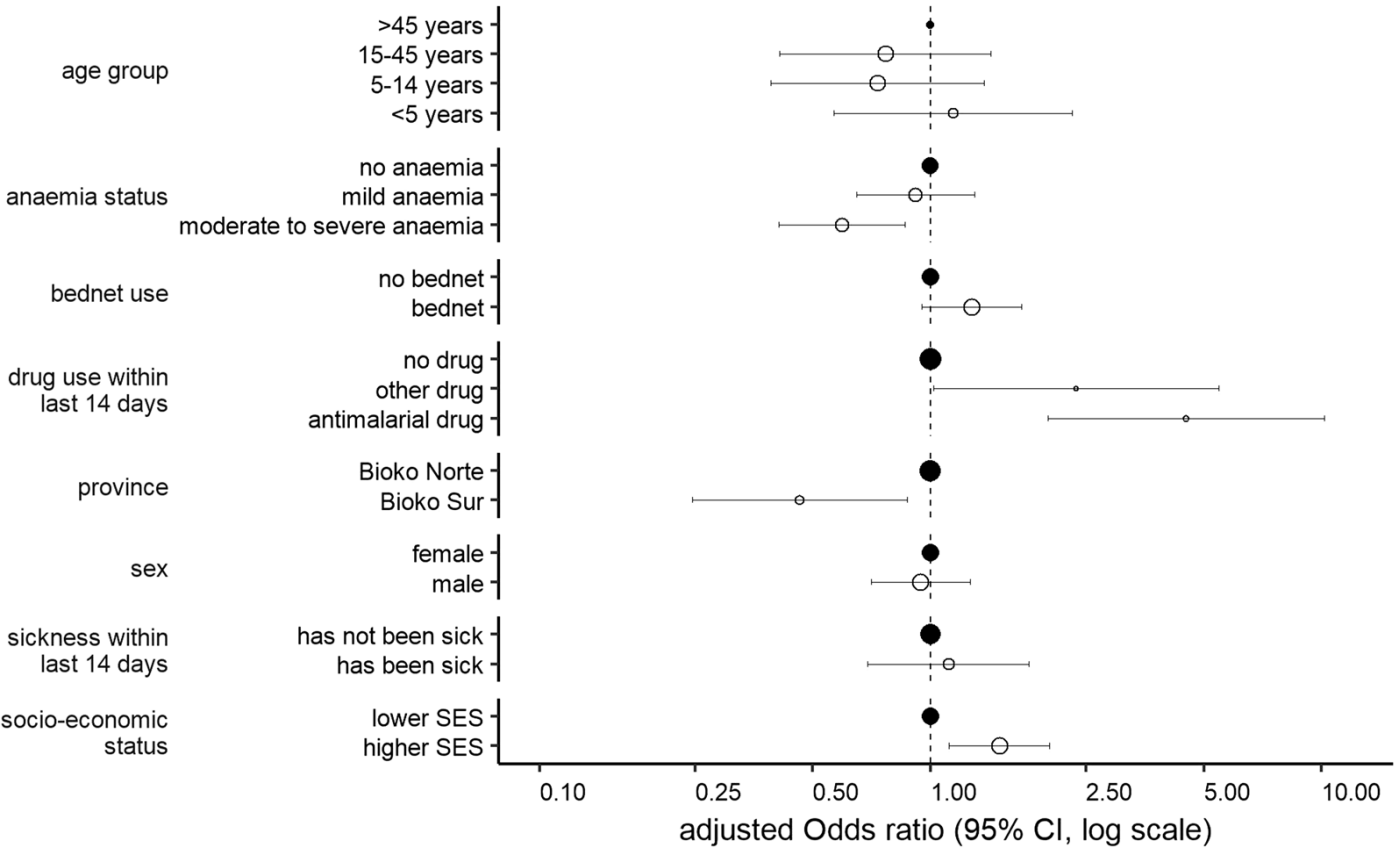
Risk factors associated with false-positive rapid diagnostic test results by multivariate logistic regression analysis. The size of the circles corresponds with the number of responses for each variable outcome. The reference group is marked by filled circles and the other groups have open circles. Higher socio-economic status (SES) included people from the 4th and 5th wealth quintiles

The rate of FP-RDTs differed across age, level of anaemia, geographical location of residence, and the SES (Additional file 1: Fig. S2). Interestingly, no study participant with a FP-RDT had a fever (> 37.5 °C) at the time of survey, while 1.6% (12/754) of those with TP-RDTs were recorded with fever. Eight variables collected during the MIS were used to identify risk factors associated with FP-RDTs through multivariate logistic regression analysis in which the outcome of the test was set as the outcome variable (Additional file 1: Table S1). FP-RDTs (n = 297) were compared to TP-RDTs (n = 754). Because sample collection was clustered within communities, community affiliation was introduced as a random effect to the model. The MIS included 299 communities, of which 201 (67.2%) were represented in the dataset. The median number of samples from a community was 3. Survey participants belonging to higher socio-economic classes (aOR 1.51 p = 0.01) had increased odds of having a FP-RDT. Participants who were reported to have been treated with an anti-malarial drug 2 weeks preceding the survey had more than four times the odds of a FP-RDT result than a TP-RDT (aOR 4.52, p < 0.001). Noteworthy, 46.6% (136/292) of the participants who had received an anti-malarial treatment in the 2 weeks preceding the survey did recall what drug they had been treated with. The majority of MIS participants (80.9%, 110/136), who reported to have received recent anti-malarial treatment, mentioned that they had received artemisinin derivates or ACT. Due to the small number of MIS participants treated with non-ACT anti-malarials, the variety of anti-malarials used within this group, and the fact that this information is self-reported, it was decided not to include any further analysis, including a breakdown into individual drugs. In contrast, moderate to severe anaemia reduced the odds of having a FP-RDT (aOR 0.60, p = 0.02). Those who reside in the rural Bioko Sur Province had also decreased odds of having a FP-RDT (aOR 0.44, p = 0.01). Age, gender, bed-net use, and reported sickness in the 2 weeks preceding the survey were not significantly associated with FP-RDTs (Fig. 4).

### The impact of asymptomatic malaria infections on anaemia status might be underestimated by FP-RDT results

**Fig. 5:**
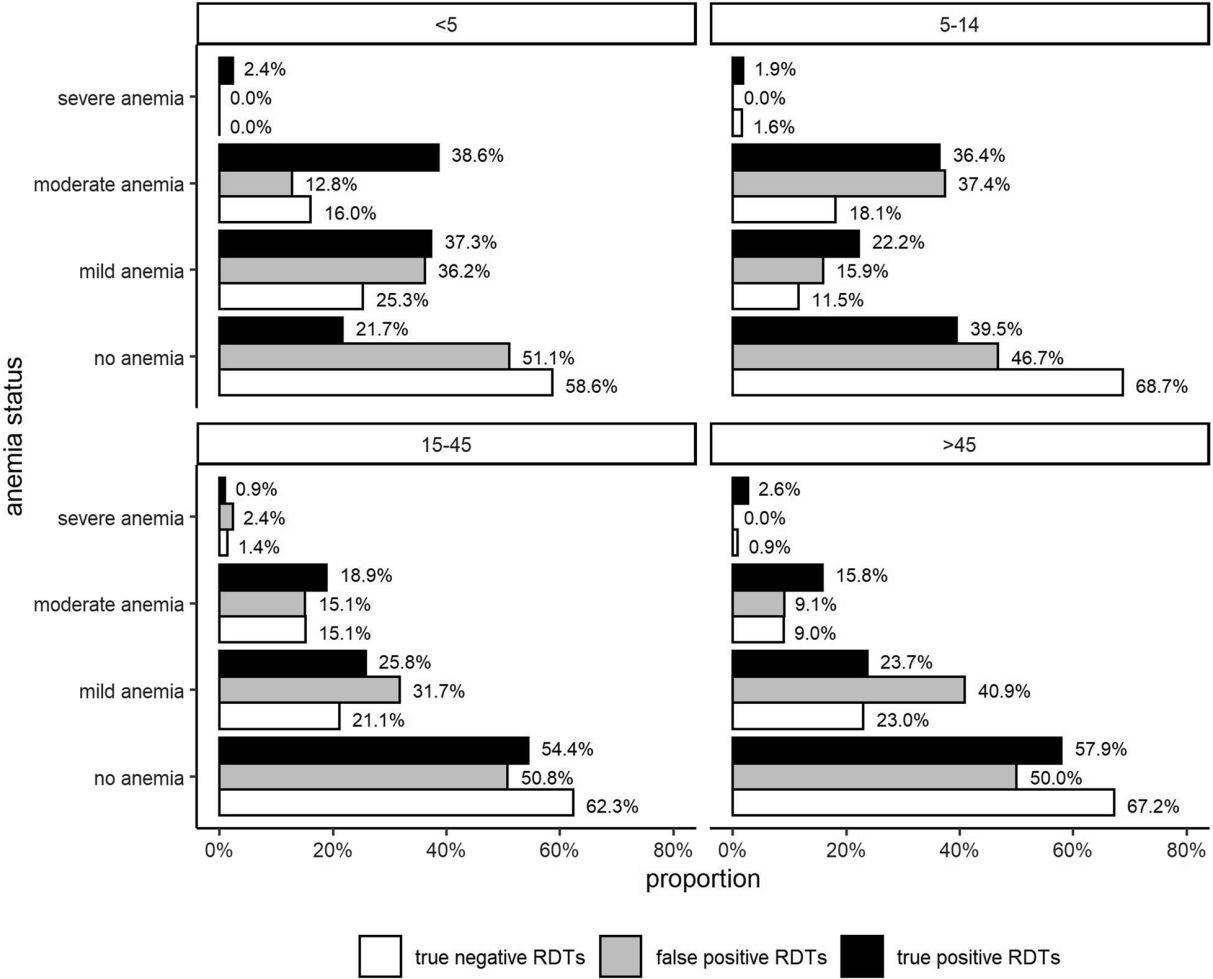
Proportion of volunteers with different anaemia status stratified by true-positive, false-positive and true-negative rapid diagnostic test outcomes. The anaemia status was stratified by age group (< 5, 5–14, 15–45 and > 45 years). For TP-RDT, FN-RDT and TN-RDT test outcomes, the proportion of all participants of each age group with no, mild, moderate or severe anaemia was calculated

It was hypothesized that high rates of FP-RDTs are likely to result in underestimating the impact of asymptomatic malaria infections on the anaemia status. Among malaria-infected children aged < 5 years, the prevalence of anaemia was 67.7% if malaria status was assessed by RDT. Proportion of anaemic children with FP-RDT result (48.9%) is similar to children with TN-RDT result (41.4%) (p = 0.85, Fisher exact test), whereas children with a TP-RDT result are more likely to suffer from anaemia (78.3%) (p = 0.0005, Fisher exact test) (Fig. 5). This significant effect is even more pronounced among children < 5 years with moderate and severe anaemia if compared to mild anaemia. Removing all FP-RDTs in this association between malaria infection status and anaemia levels in children < 5 years reveals that the association between asymptomatic malaria with moderate or severe anaemia might be even stronger. In older children and adults, the impact of FP-RDTs on assessing the anaemia status is negligible.

## Discussion

Malaria control programmes rely on continuous and systematic collection of surveillance data for decision making and resource allocation [35]. A critical measure that closely reflects malaria transmission intensity is the parasite rate, which is the proportion of the population found to carry parasites in their blood [36]. RDTs, more specifically PfHRP2-based RDTs, are the most widely used test to measure parasite rates in endemic countries and are a cornerstone of malaria control. However, diagnostic performance issues of PfHRP2-based RDTs were identified to be particularly related to limited specificity. Therefore, malaria surveillance depending solely on RDTs might profit from well-integrated quality control procedures assessing the potential impact of reduced sensitivity and specificity of the RDT used. Presented in this report is an efficient approach to assess the performance of field-deployed RDTs used for malaria surveillance based on NA extraction from the RDTs followed by qPCR analyses.

*Plasmodium* spp. NA was found in 4.7% (84/1800) of the negative RDTs and were classified as FN-RDTs. The low proportion of FN-RDTs can be explained by the low parasite densities in these asymptomatic individuals (geometric mean of 5.4 *P. falciparum*/µL) and the low amount of blood (one drop corresponds to approximately 5 µL) used as starting material for the molecular analysis. This is a certainly one of the major limitations of the approach. In a previous study conducted among asymptomatic blood donors in Malabo, PfHRP2-based RDTs showed a sensitivity of only 23.1% and more than 75% of infections had densities below 100 *P. falciparum*/μL [27]. Therefore, the true proportion of FN-RDTs in a high prevalence setting such as Bioko Island is likely to be higher than reported here.

*Plasmodium falciparum* isolates were identified with potential *pfhrp3* deletions but not a single isolate with a confirmed *pfhrp2* deletion. Given the overall high frequency of polyclonal *P. falciparum* infections in this setting (63.0% by *pfmsp1/pfmsp2* genotyping), it was assumed that if *P. falciparum*-carrying *pfhrp2* deletions exist, then they would be most likely masked by co-infecting *P. falciparum* isolates without *pfhrp2* gene deletions. Of all the samples included for final analysis, 11.1% had an increased ΔCq value for *pfhrp2* and 11.1% for *pfhrp3* amplification, indicating for the first time that there are likely *P. falciparum* strains circulating on Bioko Island carrying deletions in their *pfhrp2* and/or *pfhrp3* genes. So far, one report described *P. falciparum* strains carrying *pfhrp2* and *pfhrp3* deletions in blood samples collected on the continental region of Equatorial Guinea [37]. Since travel activity between Bioko Island and the mainland of Equatorial Guinea is high, it can be assumed that parasite strains are exchanged frequently between these locations [38]. Most importantly, blood samples with *P. falciparum* clones indicative of masked *pfhrp2* and *pfhrp3* gene deletions were recorded as PfHRP2 positive by RDT. Likely, the co-circulating *P. falciparum* clones compensate for the lack of PfHRP2 expression resulting in RDT-positive testing. In 462 clinical samples from different African countries, 7.4% (34/462) samples carried a *pfhrp2* deletion and 10.6% (49/462) a *pfhrp3* deletion, while masked *pfhrp2* and *pfhrp3* deletions were found in 3.0 and 3.2% of samples, respectively [39].

The data support the notion that in settings where polyclonal *P. falciparum* infections are common assays with the ability to identify masked *pfhrp2* and/or *pfhrp3* gene deletions should be used [40]. Importantly, to avoid false reporting of *pfhrp2* and/or *pfhrp3* gene deletions, a robust and multi-layered approach was used by which only samples with a pre-defined parasite density, successful amplification of the assays’ internal control, and additional, independent amplification of either *pfmsp1* or *pfmsp2* genes were included into the analysis.

In this study, a significant proportion of FP-RDTs were discovered. The findings are not unique to Bioko Island. In a study conducted in Tanzania, 22% of malaria-positive RDTs were negative by molecular analysis for *P. falciparum* [41]. A study performed in Guinea-Bissau reported 26% FP-RDTs [42], and in Western Kenya, approximately one-third of positive RDTs were negative by molecular detection methods for *P. falciparum* [43]. With introduction of a novel RDTs labelled as ‘ultra-sensitive’, detecting lower concentrations of the PfHRP2 antigen, the problem of FP-RDT results is expected to become greater, as already shown in a recent study [44].

The wrong positivity of RDTs based on PfHRP2 detection could be associated with recent use of anti-malarial drugs confirming previous reports [23, 45–48]. It has been well established that anti-malarial treatment leads to FP-RDT results because the PfHRP2 antigen persists in the blood days to weeks after parasite clearance [23, 45–48].

In addition, an association was found between FP-RDTs and potential access to anti-malarial drugs based on higher SES and on living in urban parts of the Island.

The impact of FP-RDTs differs greatly depending on the setting in which RDTs are deployed. In clinical settings, FP-RDTs might be less common, but the consequences are serious since wrong prescription of anti-malarials might increase risk of overlooking other life-threatening diseases causing fever [49]. In cases where RDTs are used for epidemiological surveys, a high proportion of FP-RDTs due to PfHRP2 antigen persistence might lead to an overestimation of malaria prevalence, particularly in populations with good access to anti-malarial treatment. Using RDT only as test for malaria infection status might underestimate the negative consequences of asymptomatic malaria infections on haemoglobin levels, particularly in children < 5 years of age [50].

The benefits and the challenges that come with large-scale deployment of molecular techniques for malaria surveillance in malaria-endemic regions have been discussed [51]. Alternative and non-molecular approaches such as automated malaria diagnosis using haematology analysers [52] should be further evaluated for malaria surveillance purposes. The ongoing COVID-19 pandemic has raised the awareness of the value of introducing novel methods as surveillance tools in the public health systems in Africa [53]. Building on this experience will potentially accelerate efforts to integrate sensitive and specific tools for continous, large-scale surveillance of malaria in control programmes.

## Conclusion

Malaria surveillance programmes based on RDT assessments of malaria prevalence should be strengthened by the integration of molecular epidemiological data in the same setting. These data will serve as an early warning system for (i) spread of *P. falciparum* strains evading widely used diagnostic tests; (ii) understanding overuse of malaria drugs; (iii) help with identifying fever-causing diseases beyond malaria; and, (iv) help to clarify the burden of asymptomatic malaria as a cause of severe to moderate anaemia, particularly in children < 5 years.

## Supplementary Information

**Additional file 1:** **Figure S1.** Genetic diversity of *Plasmodium* *falciparum* and *Plasmodium* *malariae* length polymorphic genes. **Figure S2.** False-positive rapid diagnostic tests as a proportion of all positive rapid diagnostic tests. **Table S1.** Multivariable logistic regression analysis of risk factors associated with false-positive rapid diagnostic tests.

## Data Availability

All data needed to evaluate the conclusions in the paper are present in the manuscript or the Additional files. Further information will be made available to interested researchers.

## Abbreviations

RDT: Rapid diagnostic test
PfHRP2/3: Histidine rich protein 2/3
ENAR: Extraction of nucleic acids from RDTs
MIS: Malaria indicator survey
RT-qPCR: Reverse transcription quantitative polymerase chain reaction
Cq: Quantification cycle
sSA: Sub-Saharan Africa
ACT: Artemisinin combination therapy
SES: Socio-economic status
NA: Nucleic acid
WHO: World Health Organization
PCA: Principal component analysis
FAM: Fluorescein
NTC: Non-template control
