# Supplementary material for: Analysis of nucleic acids extracted from rapid diagnostic tests reveals a significant proportion of false positive test results associated with recent malaria treatment
Source: Malar J. 2022 Jan 24;21:23. doi: 10.1186/s12936-022-04043-7 (PMC8785039; doi:10.1186/s12936-022-04043-7)
Supplement: Supplementary file 1 — Additional file 1: Figure S1. Genetic diversity of Plasmodium falciparum and Plasmodium malariae length polymorphic genes. Figure S2. False-positive rapid diagnostic tests as a proportion of all positive rapid diagnostic tests. Table S1. Multivariable logistic regression analysis of risk factors associated with false-positive rapid diagnostic tests. [file 12936_2022_4043_MOESM1_ESM.docx]

# **Additional files**


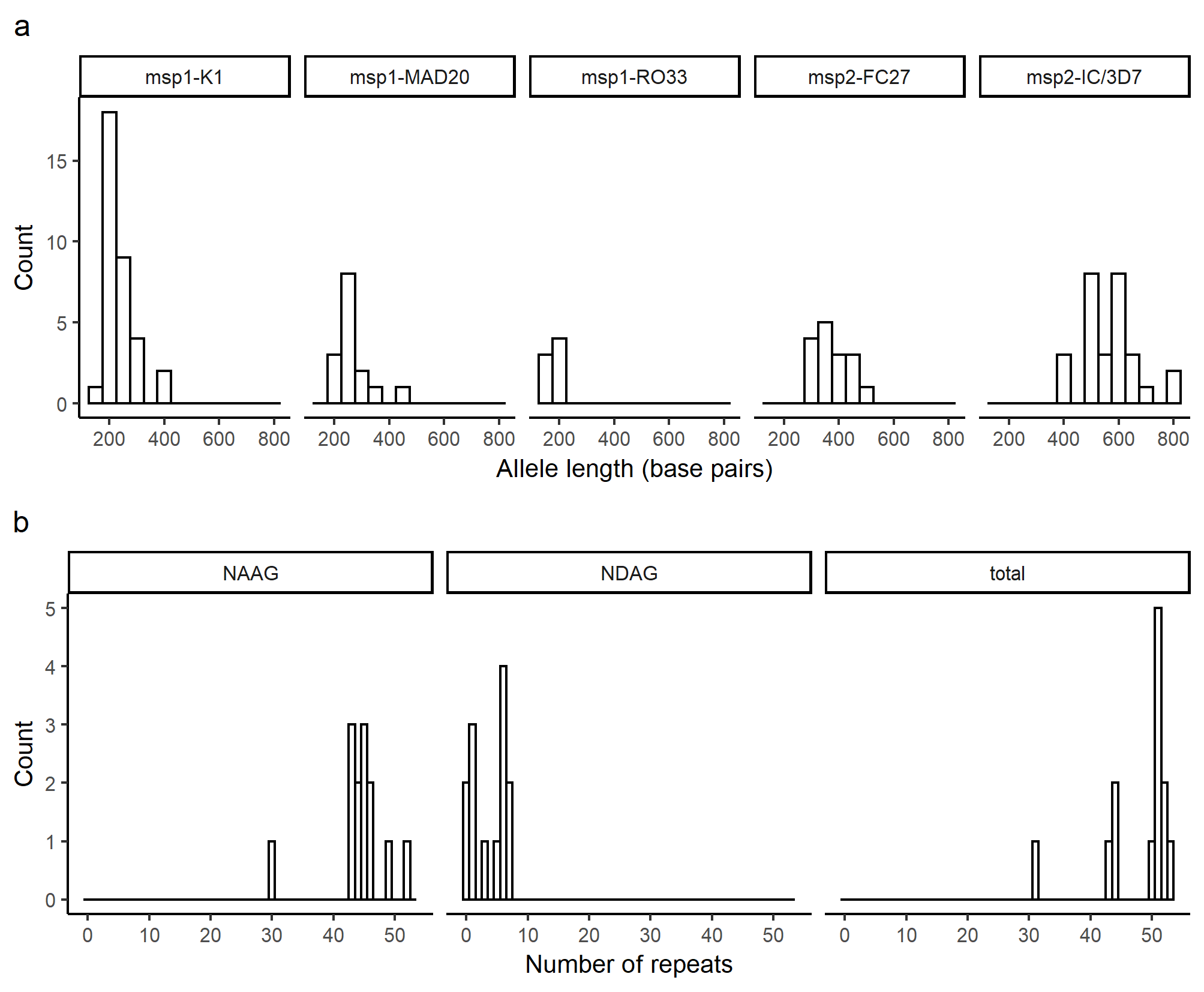


**Additional Fig. 1 Genetic diversity of *Plasmodium falciparum* and *Plasmodium malariae* length polymorphic genes**

(a) Genetic diversity of *P. falciparum* determined by size polymorphism of *pfmsp1* and *pfmsp2*. (b) Genetic diversity of *P. malariae* determined by the number of NAAG and NDAG repeats in the *pmcsp* gene.

**
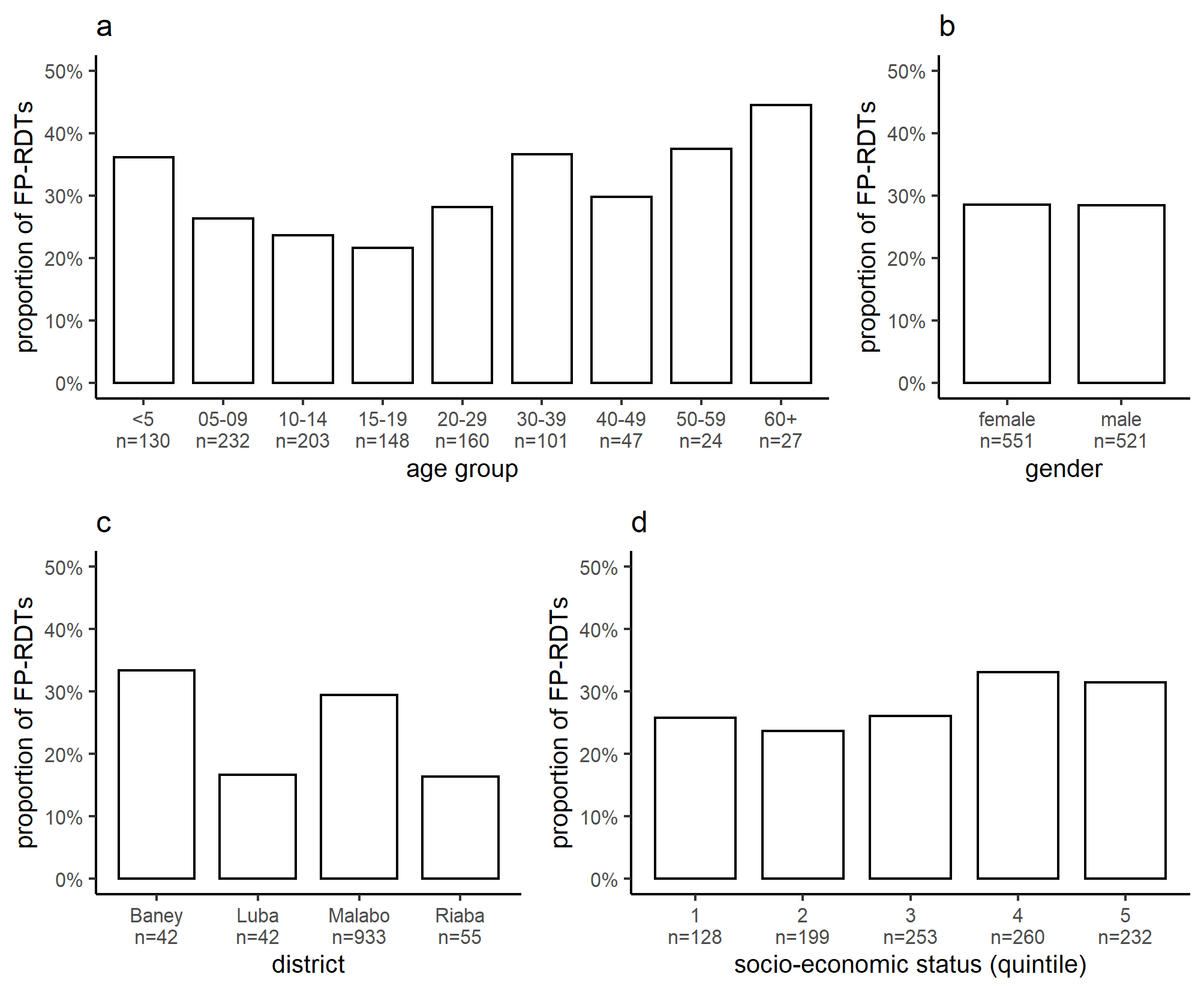
**

**Additional Fig. 2 False-positive rapid diagnostic tests as a proportion of all positive rapid diagnostic tests**

Stratified by age (a), gender (b), district (c), and socio-economic status (d). Quintile 1 refers to the lowest, while quintile 5 to the highest socio-economic status.

| Factor | Level | N | FP-RDT  N (%) | Crude OR  (95% CI) | Crude P value | Adjusted OR  (95% CI) | P (LR test) |
| --- | --- | --- | --- | --- | --- | --- | --- |
| Age group | >45 years (ref) | 59 | 21 (35.6%) |  |  |  | 0.23 |
|  | 15-45 years | 436 | 106 (24.8%) | 0.73  (0.41, 1.29) | 0.28 | 0.77  (0.41, 1.43) |  |
|  | 5-14 yea | 428 | 125 (28.7%) | 0.6  (0.33, 1.06) | 0.08 | 0.73  (0.29, 1.38) |  |
|  | <5 years | 128 | 45 (35.2%) | 0.98  (0.51, 1.87) | 0.72 | 1.15  (0.57, 2.31) |  |
| Anaemia status | Non anaemia (ref) | 484 | 147 (30.4%) |  |  |  | 0.02 |
|  | Mild anaemia | 272 | 81 (29.8%) | 0.97  (0.7, 1.34) | 0.87 | 0.92  (0.65, 1.30) |  |
|  | Moderate to severe anaemia | 295 | 69 (23.4%) | 0.7  (0.5, 0.98) | 0.04 | 0.6  (0.41, 0.86) |  |
| Bednet use | No bed net (ref) | 568 | 145 (25.5%) |  |  |  | 0.10 |
|  | Bed net | 483 | 152 (31.5%) | 1.34  (1.02, 1.75) | 0.03 | 1.28  (0.96, 1.72) |  |
| Drug use within last 14 days | No drug (ref) | 976 | 257 (26.3%) |  |  |  | < 0.001 |
|  | Other drug | 35 | 16 (45.7%) | 2.36  (1.19, 4.65) | 0.01 | 2.37  (1.02, 5.48) |  |
|  | Anti-malarial drug | 40 | 24 (60.0%) | 4.2  (2.19, 8.03) | <0.001 | 4.52  (2.01, 10.2) |  |
| Province | Bioko Norte (ref) | 955 | 282 (29.5%) |  |  |  | 0.01 |
|  | Bioko Sur | 96 | 15 (15.6%) | 0.44  (0.25, 0.78) | 0.01 | 0.46  (0.25, 0.87) |  |
| Gender | Female (ref) | 543 | 154 (28.4%) |  |  |  | 0.71 |
|  | Male | 508 | 143 (28.1%) | 0.99  (0.76, 1.29) | 0.94 | 0.95  (0.71, 1.27) |  |
| Socio-economic status | Lower SES (ref) | 566 | 139 (24.6%) |  |  |  | 0.01 |
|  | Higher SES | 485 | 158 (32.6%) | 1.48  (1.13, 1.94) | 0.004 | 1.51  (1.12, 2.03) |  |
| Sickness within last 14 days | Not sick (ref) | 870 | 227 (26.1%) |  |  |  | 0.65 |
|  | Sick | 181 | 70 (38.7%) | 1.79  (1.28, 2.5) | <0.001 | 1.11  (0.69, 1.79) |  |

**Additional Table 1 Multivariable logistic regression analysis of risk factors associated with false-positive rapid diagnostic tests**

Crude and adjusted odds ratios and their respective 95% confidence intervals were calculated based on comparison between FP-RDT and TP-RDT. Community was used as a random effect.
